# An exploration of the quality of life of people living with HIV in Greece: Challenges and opportunities

**DOI:** 10.1371/journal.pone.0266962

**Published:** 2022-04-14

**Authors:** Nikolaos Vrontaras, Katerina Myrvali, Dimitrios Kyrou, Simeon Metallidis, Olga Tsachouridou, Maria Chini, Maria Meliou, Mina Psichogiou, Dimitrios Basoulis, Anastasia Antoniadou, Konstantinos Protopapas, Periklis Panagopoulos, Vasilis Petrakis, Charalambos Gogos, Lydia Leonidou, Christina Karamanidou

**Affiliations:** 1 Institute of Applied Biosciences, Centre for Research and Technology Hellas, Thessaloniki, Greece; 2 1st Department of Internal Medicine, Medical School, ACHEPA University Hospital, Aristotle University of Thessaloniki, Thessaloniki, Greece; 3 Infectious Diseases Unit, 3rd Department of Internal Medicine "Korgialeneio-Benakeio" Red Cross General Hospital, Athens, Greece; 4 Internal Medicine and Infectious Diseases, National and Kapodistrian University of Athens, Athens, Greece; 5 Medical School, Laikon General Hospital, Athens, Greece; 6 National and Kapodistrian University of Athens, Athens, Greece; 7 Medical School, Attikon General Hospital, Athens, Greece; 8 Department of Internal Medicine, University General Hospital, Democritus University of Thrace, Dragana, Alexandroupolis, Greece; 9 Department of Internal Medicine and Infectious Diseases, University Hospital of Patras, Rio, Greece; Stellenbosch University, SOUTH AFRICA

## Abstract

**Objective:**

Improving the quality of life (QoL) of people living with HIV (PLWH) has been proposed as a new priority in HIV care. The objective of this cross-sectional, qualitative study was to explore the perspectives of PLWH in Greece regarding their QoL.

**Design:**

Twenty-four semi-structured interviews were conducted with PLWH receiving care across six HIV clinics in Greece. The thematic analysis of the transcribed interviews resulted in four themes and eleven subthemes.

**Results:**

First, fear of repercussions (e.g., stigmatization) makes PLWH reluctant to disclose their diagnosis in public settings or disclose accounting for factors like the confidant’s discretion. Second, participants are challenged by HIV’s unique biopsychosocial facets (e.g., uncertainty about symptoms) and fear for the future (e.g., a confidant revealing their HIV status without consent). Third, support received by specialist services is satisfactory in contrast to non-HIV specialist services, where significant improvements are needed to reduce stigmatization. Finally, the experiences of PLWH include contrasting elements of post-traumatic growth and an inability to accept their seropositivity (e.g., avoiding social interactions).

**Conclusions:**

Empowering PLWH in these QoL areas is greatly needed. Increasing the life expectancy of PLWH is only the initial step; their QoL needs to be secured as the next priority in HIV care.

## Introduction

In recent years, infection by the Human Immunodeficiency Virus (HIV) has been transformed from a terminal illness to a manageable, chronic condition. Advances in antiretroviral therapy have substantially prolonged the lives of people living with HIV (PLWH) [1]. Possibly because of this newfound increase in their life expectancy, PLWH are facing newly emerging challenges that remain unaddressed [1]. Ageing with HIV means that PLWH are and will continue to be experiencing lifelong health care needs.

Clinicians and healthcare systems must adjust to delivering person-centred healthcare services responding to PLWH’s needs [2, 3]. The World Health Organization (WHO) in its new Global Health Sector Strategy on HIV for 2016–2021 calls for the ‘90-90-90’ target to increase diagnosis of HIV (at 90%), increase treatment initiation among those diagnosed (at 90%) and achieve viral suppression among those treated (at 90%) [4]. A fourth goal has also been proposed: having 90% of individuals with viral suppression achieve healthy ageing with good quality of life (QoL) [2]. Introducing QoL in the HIV care cascade concretely signifies a move away from the purely biological model towards the biopsychosocial model of care [5].

A review of QoL among PLWH [6] proposed that HIV affects QoL across four main domains: 1) the physical, which entails symptoms and the disease’s impact on the activities of daily living, 2) the psychological, which refers to the individual’s beliefs, feelings, and self-concept, 3) the social, involving relationships and social contacts and, 4) the environmental, which refers to home, safety, health, and social care quality.

Specifically, while their survival rates continue to improve [2], PLWH can experience serious non-communicable diseases (e.g., kidney disease, cardiovascular disease) [2], at higher rates than the general population [4, 7]. The presence of increased comorbidities suggests that their health-related QoL is lower than that of the general population.

Moreover, the psychological and social sequelae inherent to an HIV diagnosis indicate that other aspects of PLWH’s QoL are also greatly affected. According to a cross-sectional survey [8], PLWH often report low QoL due to psychological symptoms. PLWH face challenges such as depression, anxiety, financial stress, fear of transmitting HIV to others, uncertainty about starting a family and a multitude of other everyday-life issues [2].

Furthermore, according to a review of qualitative findings, the social life of PLWH is impacted by HIV-related discrimination and social stigma, resulting in isolation, secrecy, fear, guilt, and shame [9]. Recent evidence also suggests that PLWH may experience stigmatisation and discrimination even within healthcare environments. This might result in avoiding visits to physicians, self-medicating and concealing their HIV diagnosis unless absolutely necessary [9]. Therefore, even accessing services for support can be challenging for PLWH, negatively impacting their QoL.

Specific aspects of the experiences of PLWH in Greece have been explored. A recent qualitative study looked at the adaptation of life with HIV as a process of moving towards acceptance [10]. Findings revealed that individuals were initially shocked by the HIV diagnosis; however, healthcare professionals helped them adjust to the diagnosis by providing appropriate information about the disease (e.g., physical symptoms, medication and viral load management) and empathic communication. Conscious social withdrawal was common as individuals tried to make sense of the diagnosis and come to terms with a perceived threat to their life and the possibility of dying. Finally, findings highlighted hope and social support as essential elements of an easier transition into a new life and a possible connection between improved well-being and improved physical health.

Another qualitative study focused mainly on the experience of stigma among PLWH and their coping strategies [11]. One of the most important findings concerned the ambivalence experienced towards HIV, expressed in a range of positive and negative emotions. Specifically, HIV could trigger anxiety because of social stigma. Nevertheless, HIV had become an integral part of the participants’ identity, inciting a need to be understood and be to treated with compassion.

The above studies, despite their evident significance, focused on a single aspect of life (e.g., psychological adaptation, social stigma). This risks missing areas of importance for PLWH and comes in contrast with the significance currently placed on the exploration of QoL for PLWH in its totality [2]. Widely exploring possible areas of significance regarding QoL among PLWH will contribute to the provision of enhanced, holistic, biopsychosocial care.

The present study aims to explore the perceptions of people living with HIV in Greece on their quality of life.

## Methods

This study used a cross-sectional, qualitative methodology and adopted a realist approach [12, 13]; focusing primarily on exploring the unique experiences of individuals while assuming there is no universal reality that can be generalized. Data were gathered via semi-structured interviews with PLWH who were receiving treatment in specialised HIV units in Greece. Purposive recruitment [13] was employed to explore diverse experiences of PLWH between September 2019 and November 2019. The consolidated criteria for reporting qualitative research (COREQ) [14] were used in this study (see S1 Appendix).

### Participants

The participants were 24 PLWH; 18 men and 6 women. They were receiving treatment in six specialised HIV clinics of state hospitals in major cities in Greece (Athens, Thessaloniki, Alexandroupoli, Patras). Twenty-three participants were receiving treatment as outpatients and one as an inpatient. Their reported time since diagnosis ranged from one month to twenty years (average of 7.5 years, SD = 6.1 years). To protect their confidentiality, no other demographic information was recorded.

### Material

A semi-structured interview guide was used for data collection. The interview guide was developed after an extensive literature review, where a list of possible questions to assess the participants’ experiences were identified and compiled. Open-ended questions were developed to allow interviewees to express their views fully and in-depth [13, 14]. An example question is ‘*Drawing from your experiences*, *how is health care provision for people with HIV*?’ (see S2 Appendix).

### Procedure

Healthcare professionals first approached potential participants and gave a brief description of the study and its goals. The interviewer (KM; woman, Psychologist and Psychotherapist, Research Assistant, M.Sc.) then gained informed consent from those who expressed interest in learning more about the study and then arranged for the interviews to be conducted.

A researcher with extensive experience interviewing vulnerable individuals (KM), conducted the interviews face-to-face in Greek. The interviewer had no prior relationship with the participants. When necessary, follow-up questions were also asked in order to have a more thorough understanding of the issues raised. Twenty-three of the interviews took place in an assigned physician’s office within each HIV outpatient clinic, while one interview took place in a private room at an inpatient unit. The participants’ privacy was secured and there were no other individuals present during the interviews. None of the participants refused to participate or withdrew after they had given consent.

The interviews were audio-recorded and lasted between 15 min and 65 minutes (mean 30 mins). Saturation was reached when no new themes emerged, at which point data collection concluded. KM and CK (woman, Health Psychologists, Post-Doctoral Researcher, PhD) transcribed and analysed the interviews. No formal transcription method was used, as it is not always needed for thematic analysis [12]. Microsoft Office was used to manage the data.

### Analysis

Thematic analysis was employed for the analysis of the data [12]. All interviews were read thoroughly several times to extract the meaning of each participant’s statements. Through reflecting and coding, themes developed from the data and relevant quotes were used to support each theme [12, 15]. The initial codes were grouped into eleven subthemes, which in turn were grouped into four major themes. A different member of the research team (NV; man, Health Psychologist, Researcher Associate, M.Sc.), then read the interviews and tried to match their content with the themes/subthemes. It was found that the existing themes/subthemes captured accurately the interviews’ content.

The emerging themes, subthemes, and quotes were translated from Greek into English by two members of the team (KM, NV). They were are also checked for comprehensibility by a third member of the research team (CK).

### Ethical issues

During the recruitment process, participants were informed of the nature and purpose of the study in written form; they were informed of the protection of their confidentiality and anonymity, of their right to refuse to answer questions or to remove themselves from the study. The interviews were conducted only after the participants signed a consent form.

The interview audio recordings were stored according to the EU General Data Protection Regulation [16]. Any information that could identify the participants was anonymized at the time of transcription. No participants reported any distress caused through the interview process and an informal debriefing was offered to each participant after the conclusion of their interview.

Ethical approval for this research study was granted through the Research Ethics Committee of the Institute of Applied Biosciences at the Centre for Research and Technology Hellas as well as through the Research Ethics Committee of each of the six participating hospitals.

## Results

Following the analysis of the interviews conducted with PLWH, the resulting four major themes were summarized with representative quotes for each of the eleven subthemes in S3 Table in S3 Appendix. Overall, PLWH talked about the threats to their QoL and the importance of maintaining their QoL. Participants’ perceptions of their QoL are reported below.

Notably, the four domains of QoL impacted by HIV as described by Basavaraj and colleagues [6] constitute a useful framework for interpreting the present findings. Markedly, the analysis was inductive; themes were developed from the interviews, not from any theoretical model. Nevertheless, broad comparisons between Basavaraj and colleagues’ framework and this study’s themes could be made. Specifically, the ‘Disclosure’ theme relates to the social domain, as it refers mainly to thoughts and experiences of disclosing the diagnosis to others. The ‘Impacted in the Present and Fearing the Future’ theme entails concerns about physical and biological aspects of HIV, like experienced symptoms or medication-taking. ‘Healthcare Services’ is a significant aspect of the environmental domain. Lastly, ‘Two paths lie ahead’ appears to relate to the psychological domain, since some individuals adapt better in certain QoL areas compared to others.

### Seropositivity disclosure

Immediately after receiving the diagnosis of HIV, most participants, despite being overwhelmed, needed to decide whether they would reveal their HIV status and to whom. This was described as a complex decision, characterized by internal conflict. One participant said: “*During our lifetime*, *we have to come out of the closet twice*. *Once regarding our sexuality*, *even if it is just to ourselves*, *and a second regarding this [HIV]*.*”* (20). Participants’ stance regarding disclosure went through transitions over time; at first, they reported disclosing only to a few people, but eventually, they arrived at a point of disclosing more freely and according to relevance. The participants had mixed experiences once they did make such a disclosure.

#### Conditional disclosure

Participants reported feeling that being seropositive is personal information; that it could not and should not be shared with everyone. Nevertheless, some said that although they would not share their diagnosis unless it was relevant, they would not actively lie about it. For example, they would not spontaneously share it in an eye exam, but they would share it with a gynaecologist. Before the disclosure, they reported considering the confidant’s personality, integrity, HIV knowledge and ability to conceal the information from others. Also, participants mentioned that they tended to disclose to people who could cope with and manage the news well. Participants tended to disclose to people they felt close to, sometimes including partners, family members, friends, or physicians. In certain cases, they shared their diagnosis to request support or as a means of processing their new diagnosis. In other cases, they disclosed their diagnosis to keep others safe (e.g., current sexual partner, people they share accommodation with).

#### Reasons for non-disclosure

Almost all participants reported they would not disclose their diagnosis to certain individuals (e.g., their employer). Two individuals went as far as saying they had never shared their status with anyone and did not intend to do so. In certain cases, they would not share their diagnosis because they did not want to cause distress or worry to close family or friends. Most hoped to avoid potential challenges they would face, like uncomfortable questions about their private lives or social stigma (e.g., rejection, isolation). Individuals also feared that the person they shared it with might inadvertently disclose it with others. Finally, participants refrained from sharing their status with prospective casual sexual partners due to fears of rejection, even while ensuring to take all necessary precautions. A few participants described their experience as facing an impossible dilemma; they must choose between feeling dishonest or being rejected.

#### Disclosure experiences

In certain cases, participants who proceeded to reveal their HIV status received very warm and positive reactions from others; they reported feeling accepted. They described that these experiences enabled them to come to terms with the disease, since they felt they were not facing it alone. Participants’ disclosure also transformed the relationship with the person they disclosed to and made it stronger, closer and more meaningful.

In other cases, participants had negative disclosure experiences due to being rejected and ostracized, which led to feelings of shame, guilt, depression, anxiety, loneliness, and abandonment. In certain cases, their psychological status impacted their health behaviours, namely regarding seeking medical care as well as their commitment to taking their medication.

### Challenged by the present and fearing the future

Most participants described living well with HIV. Nevertheless, they still had to manage its impact, be it actual challenges or potential fears and threats. One participant focused on the difference between trying to survive and trying to live: *“The only certainty is that you can live with HIV*, *that is the easy part*. *The rest is difficult” (54)*.

#### Present challenges

Participants described numerous challenges they were facing because of HIV. For example, they talked about putting in great effort in consistently taking their daily medication and thus managing HIV. Most must miss workdays to attend clinic appointments, or they felt they should take extra care during the winter months to avoid becoming ill. Some behaved outside social norms when attending social events (e.g., refrained from eating or drinking, avoided handshakes) because they felt this would reduce the risk of infecting others; this made participants very self-conscious of their seropositivity. Participants described being perplexed by new symptoms when they were unable to discern any clear causal attributions for the symptoms’ origins. They would wonder if what they were experiencing was a result of HIV, its treatment, a new disease or even a normal part of ageing. Further, they had difficulties coming to terms with their diagnosis and its chronic nature (e.g., saying they could never be ‘*cured*’), emphasizing their hopes for a complete cure. Few talked about still experiencing difficulties accepting the conditions under which they were infected (e.g., someone intentionally infecting them).

#### Fearing the future

Most participants talked about HIV increasing their fear, uncertainty, and concern around their future QoL. The most frequent fears were: disclosure of their HIV status by others, interpersonal fears (e.g., being rejected by potential partners); and, fear of spreading the virus to others. The latter was particularly true during intimate times when participants’ awareness of HIV was said to be especially vivid, almost like a distinct ‘*presence*’. Certain participants were anxious they might contract another sexually transmitted disease (STD) during a sexual encounter with someone new. The participants further feared the consequences of missing even one dose of their daily medication. Participants were similarly concerned about HIV making them more susceptible to other health conditions or rendering them vulnerable to health professionals’ maltreatment or neglect should they end up in a care facility.

### Receiving health care

The specialised HIV healthcare services were reported to be vital in managing their condition but also sustaining their overall physical and emotional health. Participants shared their perceptions of the HIV units and the role of the medical staff. Moreover, they shared negative experiences from their contact with the other healthcare services.

#### The support of the specialised HIV units

All participants expressed deep satisfaction with the healthcare services received in the specialised HIV units, derived from the compassionate, welcoming and accepting way they were received. Participants expressed their gratefulness to the medical staff for making them feel safe, supported, cared for. They also acknowledged the challenges the units have to overcome to provide care. Nevertheless, they stated that the units were well-equipped to respond to their needs.

#### The complexity of the physician’s role

Most participants indicated that the physicians’ role is complex and multilateral. They were also expected to play the roles of a friend, a parent, and a person who inspires and empowers them. Furthermore, due to the participants’ marginalization from other services, physicians helped and supported them with issues not related to HIV. They talked warmly about their confidence in their physicians. They said that their commitment to taking medication originates from the trust they have placed in their physicians. However, participants also talked about how sometimes they felt their physicians did not have enough time for them.

#### Negative experiences with healthcare

Participants’ negative experiences mostly related to interactions they had with professionals outside the specialised HIV clinic. They reported that as soon as they shared their diagnosis with other healthcare professionals, they were faced with ignorance, prejudice, and rejection. For this reason, many participants were unwilling or wary of visiting other professionals outside the HIV clinics and did not always disclose their HIV status to them. They reported trying to resolve their health concern either by going to the pharmacy or when possible by visiting the HIV unit.

#### Two paths lie ahead

Being diagnosed with HIV was reported as a major and life-changing experience. According to the findings, two paths emerged, one of acceptance and growth and another of an inability to move forward; one participant said: “*you can turn this [HIV] into a wall or a stepping stone”* (54). This presents two diametrically opposite ways of living with HIV. Notably, participants talked about having grown in certain aspects of their lives while at the same time feeling unable to accept others. They further talked about having a ‘toolbox’ of resources that helps them improve their lives.

#### Stepping stone

Most participants felt they had adjusted well to their new reality and said that they are living good lives even while having to manage a chronic condition. They said they have not seen HIV as an obstacle or a burden, and they thought that it did not deprive them of experiences, relationships, setting goals or dreams. Accepting the disease was reported as being pivotal in this transformation. Participants described coming to terms with the diagnosis through challenging previously held beliefs and changing their way of living. They mentioned that HIV has helped them make positive changes in their lives and experience psychosocial growth. For example, they recounted that it has allowed them to enjoy each moment, to feel fulfilled and content and to make better life decisions. Thus, they reported finding new personal meaning in living with HIV.

#### Hitting a wall

For a minority of participants, many aspects of their lives were affected negatively. They talked about being unable to accept their diagnosis. They reported that, for at least certain aspects of their lives, HIV has kept them from living their life to its fullest. For example, a participant’s social interactions were greatly reduced; others refused to form any sexual relationship while few have not even allowed others to touch or hug them. One participant seemed to have a negative relationship with their own body, feeling disconnected from it, feeling ‘contaminated’ and reporting that their body did not belong to them.

#### Toolbox

Each participant reported having a toolbox of recourses they used. These included the presence and support of professionals (e.g., physicians, nurses, mental health professionals), significant others (e.g., family members, partners, friends) or seropositive peers. Their physicians’ support was described as very important to them. Participants talked about relying on adhering to their antiretroviral medication as a way of protecting and reassuring themselves. Notably, they suggested that their significant others would also benefit from support. Furthermore, the participants’ personality, beliefs, ability to self-care and healthy lifestyle were reported as useful recourses to draw from. Sometimes, they needed not to talk or even think about HIV. One participant described their duty to address their medical needs as: “*doing what needed to be done*” (18).

## Discussion

This study aimed to explore the perspectives of PLWH in Greece on their quality of life. The findings highlight four major themes: 1) first, the significance of PLWH’s disclosure of their HIV status, the conditions for disclosure and their different disclosure experiences,2) second, the challenges and fears resulting from an HIV diagnosis or illness and symptoms management,3) third, their mixed experiences with healthcare services, including the role of healthcare professionals, 4) finally, the different trajectories participants’ lives took, including how they coped from the moment of diagnosis onwards.

Participants appeared to put much thought into disclosing their HIV status to others. Present and past findings [11] suggest that PLWH cope with stigma either through concealing or through giving false information about their general health. PLWH frequently experience difficulties with social interactions, sometimes due to avoidance of sexual relationships, rejection by potential partners, fear of contracting another STD or transmitting HIV to others [17]. Furthermore, PLWH with social difficulties tended to be less resilient, with poorer QoL and mental health [18]. The current study indicates that loneliness can sometimes result from a negative disclosure experience or being unable to move on and ‘hitting a wall’. Another study [10] proposed that social isolation is sought as part of a journey towards acceptance. Present findings indicate that outcomes of disclosure experiences could potentially affect individuals’ acceptance of their HIV status.

The present study also revealed many challenges and fears related to the physical aspects of HIV infection and treatment. In keeping with the literature, PLWH worried about contracting another infection; this fear can negatively affect their well-being [18]. Other examples included fears about the future and a deterioration of their physical health. Similar aspects of medical uncertainty and uncertainty about the future were shown to impact the mental health of PLWH [18]. Moreover, participants stressed that their main concern and uncertainty was not about surviving HIV but about living with HIV. Similarly, previous research has investigated concerns of PLWH around ageing and the changes it may bring [18].

Experiences are mixed regarding the healthcare environment for PLWH in Greece; specialist services were felt to be supportive, while non-HIV specialist services were reported as needing improvement. Previous studies have explored the stigma and marginalization that can be found in non-specialist services [17]. Difficulties in transitioning between the different services and gaps in these services for PLWH have likewise been found [3, 19], meaning that PLWH have to balance who would best meet their needs and help them maintain their QoL while having to cope with the possibility of a negative experience.

Similar to previous studies, the present findings include both negative aspects of living with HIV (e.g., stigma, being unable to move on) [11], but also post-traumatic growth [20]. PLWH try to cope using different strategies (e.g., managing the symptoms, accessing support from family or friends). Models of how an individual with HIV may move towards accepting HIV have been proposed [10]. However, not everyone may reach that point of acceptance. Similarly, their acceptance may not be universal and across all aspects of living with HIV. Moreover, HIV stigma (internalized or external) might keep people from moving on [11]. Therefore, QoL among PLWH is subject to successfully overcoming the challenges they face.

### Strengths, limitations, and future directions

This is one of the few studies on this issue conducted in Greece. It has examined a broader range of the HIV experience, aiming to perform an in-depth exploration of different contributors to QoL. The large number of participants has allowed the collection of diverse experiences, demonstrating that PLWH are trying to manage threats to their QoL and are experiencing growth because of this process.

However, this approach was unable to capture the possibly varying experiences of individuals who had been receiving treatment for different lengths of time, for example, people earlier in their treatment may potentially have more or less physical health symptoms. Future research could aim to identify and explore these and how people adapt over time, along with predictors and individual differences in the challenges to the QoL and coping strategies utilized to mitigate them while accounting for treatment duration.

It can be inferred that offering psychological support would allow PLWH to better adapt to living with HIV and build on their strengths and support in managing any difficulties. Because of the unique challenges PLWH face, the need for personalized care and interventions is highlighted. The care of PLWH should also empower them through education on the following key areas: (1) How HIV is transmitted, (2) What it means when HIV is untraceable, (3) Physical symptoms, (4) Risks regarding other infections, (5) Disclosure, (6) Coping with someone disclosing their seropositivity without consent, (7) Managing social relationships, (8) Stigma, (9) The progression of HIV, (10) Ageing with HIV. Systemic changes across services should be promoted to educate healthcare professionals around providing care for PLWH, reduce stigma and address gaps in services.

Overall, this study supports the importance of adding QoL as the ‘fourth 90%’ [2] and moving HIV clinical care towards the biopsychosocial model of care [5].

## Supporting information

S1 Appendix COREQ 32item checklist. (DOCX)

S2 Appendix Interview schedule. (DOCX)

S3 Appendix Themes, subthemes and representative quotes. (DOCX)

S4 Appendix Greek-language abstract. (DOCX)

## Abbreviations

PLWH: People living with HIV
QoL: Quality of life
