# Supplementary material for: An exploration of the quality of life of people living with HIV in Greece: Challenges and opportunities
Source: PLoS One. 2022 Apr 14;17(4):e0266962. doi: 10.1371/journal.pone.0266962 (PMC9009608; doi:10.1371/journal.pone.0266962)
Supplement: S1 Appendix — (DOCX) [file pone.0266962.s001.docx]

# S1 APPENDIX - COREQ 32item Checklist

| **No. Item** | **Guide questions/description** | **Reported on Page #** |
| --- | --- | --- |
| **Domain 1: Research team and reﬂexivity** | | |
| 1. Interviewer/facilitator | Which author/s conducted the  interview? | 6, 17 |
| 2. Credentials | What were the researcher’s credentials? | 6-7 |
| 3. Occupation | What was their occupation at the time of the study? | 6-7 |
| 4. Gender | Was the researcher male or female? | 6-7 |
| 5. Experience and training | What experience or training did the  researcher have? | 6-7 |
| 6. Relationship with participants established | Was a relationship established prior to study commencement? | 6 |
| 7. Participant knowledge  of the interviewer | What did the participants know about  the researcher? | 6 |
| 8. Interviewer  characteristics | What characteristics were reported  about the interviewer/facilitator? | 6-7 |
| **Domain 2: study design** | | |
| 9. Methodological  orientation and Theory | What methodological orientation was  stated to underpin the study? | 5 |
| 10. Sampling | How were participants selected? | 5 |
| 11. Method of approach | How were participants approached? | 6 |
| 12. Sample size | How many participants were in the  study? | 5 |
| 13. Non-­‐participation | How many people refused to participate  or dropped out? Reasons? | 6 |
| 14. Setting of data collection | Where was the data collected? | 6 |
| 15. Presence of non-­‐  participants | Was anyone else present besides the  participants and researchers? | 6 |
| 16. Description of sample | What are the important characteristics  of the sample? | 5 |
| 17. Interview guide | Were questions, prompts, guides provided by the authors? | 5-6, S2 Appendix B |
| 18. Repeat interviews | Were repeat interviews carried out? | No |
| 19. Audio/visual recording | Did the researcher use audio or visual recording to collect the data? | 6 |
| 20. Field notes | Were ﬁeld notes made during and/or  after the interview? | No |
| 21. Duration | What was the duration of the interviews | 6 |
| 22. Data saturation | Was data saturation discussed? | 6 |
| 23. Transcripts returned | Were transcripts returned to  participants for comment and/or correction? | No |
| **Domain 3: analysis and ﬁndings** | | |
| 24. Number of data coders | How many data coders coded the data? | 7,17 |
| 25. Description of the  coding tree | Did authors provide a description of the  coding tree? | 7-14, S3 APPENDIX C |
| 26. Derivation of themes | Were themes identiﬁed in advance or  derived from the data? | 7 |
| 27. Software | What software, if applicable, was used to manage the data? | 6 |
| 28. Participant checking | Did participants provide feedback on  the ﬁndings? | No |
| 29. Quotations presented | Were participant quotations presented to illustrate the themes/ﬁndings? Was  each quotation identiﬁed? | 8-14, S3 APPENDIX C |
| 30. Data and ﬁndings  consistent | Was there consistency between the data  presented and the ﬁndings? | 7 |
| 31. Clarity of major themes | Were major themes clearly presented in the ﬁndings? | 8-14, S3 APPENDIX C |
| 32. Clarity of minor  themes | Is there a description of diverse cases or  discussion of minor themes? | 8-14, S3 APPENDIX C |
