# Supplementary material for: An exploration of the quality of life of people living with HIV in Greece: Challenges and opportunities
Source: PLoS One. 2022 Apr 14;17(4):e0266962. doi: 10.1371/journal.pone.0266962 (PMC9009608; doi:10.1371/journal.pone.0266962)
Supplement: S2 Appendix — (DOCX) [file pone.0266962.s002.docx]

# S2 APPENDIX – Interview Schedule

1. *If you would like, I was thinking we could start by talking a bit about your so far experience with HIV, starting at the time of your diagnosis.*

- How did you find out you were seropositive?
- How was the diagnosis delivered to you?
- What are the things that went through your mind at the time?

1. *Some people experience difficulties adjusting to the HIV diagnosis. What has your experience been?*

- Did you feel you were affected either emotionally or mentally? If so, would you like to tell me more about it?
- Are there any emotions that you felt where more prominent at the time?
- Has this changed over time? If so, would you like to tell me more about it?

1. *People living with HIV sometimes chose to disclose their diagnosis to others while at other times they do not. Would you like to tell me more about what has this been for you?*

- Do you remember any particular instances where you shared you HIV status and it went really well?
- What about the opposite? Times where it did not go so well.
- What was it that made the disclose experience go well or not so well?

1. *Do you feel your seropositivity has affected your social life? If so, would you like to describe to me in what way?*

- Relationship with family, friends, partners or co-workers?
- Do you feel it affects any new relationships you might develop?

1. *Similarly, people sometimes experience changes in their physical health. Does this hold true for you?*

- Have you experienced any symptoms from HIV or its treatment?
- How much of an impact these have on you and your day-to-day living?
- How are you able to manage these?

1. Having a chronic condition, like HIV can sometimes mean that the individual needs to adjust some of the things they do in their everyday life. Have you noticed any such changes?

- How would you say having HIV affects your everyday activities?
- Would you say this diagnosis has changed how you see yourself?
- What are your thoughts and feelings around being seropositive?
- How would you expect these to change in the future?

1. *Do you feel this diagnosis has affected your sexual relationship or practices?*

- Forming new relationships?
- Using a condom?
- Number of casual partners?

1. *Drawing from your experiences, how is health care provision for people with HIV?*

- Could you give a few examples of the time of services the HIV clinic is offering and what you think of them?
- Would you like to tell me a bit about your relationship with your physician?
