# Supplementary material for: An exploration of the quality of life of people living with HIV in Greece: Challenges and opportunities
Source: PLoS One. 2022 Apr 14;17(4):e0266962. doi: 10.1371/journal.pone.0266962 (PMC9009608; doi:10.1371/journal.pone.0266962)
Supplement: S3 Appendix — (DOCX) [file pone.0266962.s003.docx]

| S3 APPENDIX - Themes, Subthemes and Representative Quotes |
| --- |
| **Seropositivity Disclosure**  The focus of this theme is the social aspects of disclosing to others. This includes under which conditions they would or would not disclose and what might happen when they do disclose their serpopositivity. |
| **Conditional disclosure**  *“It is personal information; when you feel ready to share it, share it only with those you want to. You don’t need to disclose it wherever you go, because it really is something very-very personal.” (24)*  *“I was 100% certain and without any doubt that she would understand. This woman works in this field; in a haematology department. It’s in her nature, she is not someone who might spread such information; she is bound by confidentiality.” (53)* |
| **Reasons for non-disclosure**  *Because people know nothing of it [HIV], it is still associated with great stigma, it’s like I didn’t know before I was diagnosed. I have now learned, even if it’s not by my own choosing. When someone hears this word [HIV], they will be terrified. They might not speak to you. You might lose contact with acquaintances, friends and close one; your family might not support you. I am terrified someone might find out. “(33)* |
| **Disclosure experiences**  *“We came closer. They came closer to me. They are treating me well. I am not sure what this says about them, but I am infinitely grateful for the way they are with me and they stand by me … They said ‘so now, what can WE do about it? ’ I am not alone.” (24)*  *“For me, the day I was diagnosed was traumatic not so much due to the diagnosis but because of what followed. After I returned home, I was kicked out of the house due to the [HIV] diagnosis. I was so shocked by that and as a result, I forgot about the diagnosis. I only went to the doctor months later … My mother looks at me and asks ‘what are you doing? What happened to you?’ She takes the piece of paper [doctor’s note] and she asks me what is this? I let her take it, she reads it, I tell her about it too …. And then I took the suitcases they prepared for me and I left” (31)* |
| **Challenged by the Present and Fearing the Future**  This theme portraits the impact and challenges the participants presently face and the fears and uncertainties they hold about the future. |
| **Present challenges**  “*Since it is discrimination it is affecting an individual, it is degrading them, it is offending them, it’s irrelevant if this person has disabilities or is homosexual or anything else, the feelings and the pain are all the same.”* (17)  “*On a personal level, there is a 99% chance that if your boyfriend hears about it, then he is gone, the same for friends. They may feel sorry for you at first too. As far as to your health is concerned, there are difficulties there for sure as when you visit a hospital and tell them you are seropositive, my experience has been that they would have to have extra precautions or whatever. In any case, it’s a problem no matter how you look at it. So, I don’t know how you can deal with it. Maybe not thinking about it? Even if you wanted to, taking your medication every day keeps reminding you!”* (23) |
| **Fearing the future**  *“It is one of my greatest fears, surely I do not want anyone to find out about it [seropositivity]. Ever.” (33)*  *“Although they say I am undetectable and untransmittable, at the back of my mind, there is a constant thought … Yes, it scares me, it scares me … No one has to suffer and go through what I have been through. And obviously I want to stop the spread of the virus.” (22)*  *“What could happen to me when I grow old. It scares me … no one knows if they are going to pass on when they are young or old, but [I am scared of] how I might be treated if I am going to be admitted to an institution or a hospital.” (44)* |
| **Receiving Health Care**  This theme gives prominence to two opposite experiences with healthcare services. The supportive specialist services and the sometimes-challenging non-specialist services. The role of the HIV specialised is also described as multi-faceted and complex. |
| **The support of specialized HIV units**  *“Ok, what can I say now! Without them, we would not be alive. How can I put it? Their service is great! Not only concerning the disease but for me personally, the staff here helped me a lot. Especially, at first, when I was hospitalized, everyone was perfect, the best; my doctor is the best. What can I say? I owe them many, many, many thanks and much love. All the nurses and everyone has been very polite, kind. They did not show me any pity: ‘oh she has HIV, let’s avoid her’.” (33)* |
| **The complexity of the physician’s role**  *“She is not only my doctor, running tests and prescribing medication. She is the doctor of my soul, a lot of times we talk about these things. She is my mother, my friend.” (54)*  *“I would like to have spent more time with my doctor, to speak with her in greater detail, to know exactly which tests I have taken. It would not only be safer but it would also possibly reassure me more”. (12)* |
| **Negative experiences with healthcare**  *“We decided to have a child. We were told that due to our age it would be best to consider IVF [In Vitro Fertilization]. We went to a centre that is supposed to be one of the best for IVF. The moment I said I am HIV positive, the doctor started saying absurdities, it was as if I had taken a shot at him with a gun. Basically, he did not want to work with us and he was using numerous irrelevant excuses.” (54)* |
| **Two Paths Lie Ahead**  This theme concerns areas where participants use HIV as a stepping stone for personal growth while on other areas, they might be unable to move on and are hitting a wall. Participants also talked about different ways of coping. |
| **Stepping stone**  *“It only affected me positively, since I learned from this. The hard way true, but I learned to rely on myself, to look after myself more, to protect myself, to take care of myself. It helped me with regards to the way I am with people. In case you were wondering in what way; I became more careful in the way I offer myself and open up compared to the way I was” (31)* |
| **Hitting a wall**  *“Let us make no mistake, condoms can break. Since this is on my mind, I cannot start a relationship anymore. I will need to say something and it is much too difficult. Yes, because in order to disclose, you have already done things, you have progressed. You are already a liar. It’s not like for example, I meet you and I tell you I am HIV positive and we take it from there. I get to know you better after seeing certain things, the other person though should be aware. You have already hidden something and thus, the whole thing starts on the wrong foot and I do not want this.” (52)* |
| **Toolbox**  *“I always had close ones who were fully supportive of me. I never doubted that. I believed that no matter what happens to me, they will always stand by me.” (42)* |
