# Supplementary material for: An exploration of the quality of life of people living with HIV in Greece: Challenges and opportunities
Source: PLoS One. 2022 Apr 14;17(4):e0266962. doi: 10.1371/journal.pone.0266962 (PMC9009608; doi:10.1371/journal.pone.0266962)
Supplement: S4 Appendix — (DOCX) [file pone.0266962.s004.docx]

# S4 APPENDIX – Greek-Language Abstract

**Περίληψη**

**Στόχος**:Η βελτίωση της ποιότητας ζωής ατόμων που ζουν με HIV έχει προταθεί ως η νέα προτεραιότητα στην υγειονομική τους περίθαλψη. Ο σκοπός αυτής της ποιοτικής μελέτης ήταν η διερεύνηση των απόψεων των ατόμων που ζουν με HIV στην Ελλάδα σχετικά με την ποιότητα ζωής τους.

**Μεθοδολογία**:Διεξήχθησαν είκοσι τέσσερις ημιδομημένες συνεντεύξεις με άτομα που λαμβάνουν φροντίδα για τον HIV σε έξι κλινικές στην Ελλάδα. Η θεματική ανάλυση των απομαγνητοφωνημένων συνεντεύξεων οδήγησε σε τέσσερα θέματα και έντεκα υποθέματα.

**Αποτελέσματα**:O φόβος για τις συνέπειες (π.χ. στιγματισμός) καθιστά τους συμμετέχοντες απρόθυμους να μοιραστούν την διάγνωσή τους. Oι συμμετέχοντες λόγω του HIV αντιμετωπίζουν ιδιαίτερες βιοψυχοκοινωνικές δυσκολίες (π.χ. αβεβαιότητα σχετικά με τα συμπτώματα) και φόβο για το μέλλον (π.χ. να αποκαλυφθεί η διάγνωση χωρίς την συγκατάθεση τους). H στήριξη που λαμβάνουν από τις εξειδικευμένες στον HIV υπηρεσίες είναι ικανοποιητική, σε αντίθεση με άλλες υπηρεσίες, που χρειάζονται σημαντικές βελτιώσεις. Τέλος, οι συμμετέχοντες βιώνουν αντικρουόμενα μεταξύ τους στοιχεία, όπως δυσκολία αποδοχής της οροθετικότητάς τους (π.χ. αποφυγή κοινωνικών αλληλεπιδράσεων) αλλά και μετατραυματική ανάπτυξη.

**Συμπεράσματα**:Η ενδυνάμωση των ατόμων που ζουν με HIV στις παραπάνω θεματικές είναι σημαντική. Η αύξηση του προσδόκιμου ζωής είναι μόνο το αρχικό βήμα. Η ποιότητας ζωής χρειάζεται να διασφαλιστεί ως η επόμενη προτεραιότητα στην περίθαλψη τους.
